# Sorption of Organic Pollutants by Humic Acids: A Review

**DOI:** 10.3390/molecules25040918

**Published:** 2020-02-19

**Authors:** Simeone Chianese, Angelo Fenti, Pasquale Iovino, Dino Musmarra, Stefano Salvestrini

**Affiliations:** 1 Department of Engineering, University of Campania “Luigi Vanvitelli”, Via Roma 29, 81031 Aversa (CE), Italy; simeone.chianese@unicampania.it (S.C.); angelo.fenti@unicampania.it (A.F.); dino.musmarra@unicampania.it (D.M.); 2 Environmental Technologies, University Spin Off of University of Campania “Luigi Vanvitelli”, Via Vivaldi, 43, 81100 Caserta, Italy; pasquale.iovino@unicampania.it; 3 Department of Environmental, Biological and Pharmaceutical Sciences and Technologies, University of Campania “Luigi Vanvitelli”, via Vivaldi 43, 81100 Caserta, Italy

**Keywords:** humic acids, sorption, organic pollutants, sorption isotherm, kinetic modelling

## Abstract

Humic acids (HA) are promising green materials for water and wastewater treatment. They show a strong ability to sorb cationic and hydrophobic organic pollutants. Cationic compounds interact mainly by electrostatic interaction with the deprotonated carboxylic groups of HA. Other functional groups of HA such as quinones, may form covalent bonds with aromatic ammines or similar organic compounds. Computational and experimental works show that the interaction of HA with hydrophobic organics is mainly due to π–π interactions, hydrophobic effect and hydrogen bonding. Several works report that sorbing efficiency is related to the hydrophobicity of the sorbate. Papers about the interaction between organic pollutants and humic acids dissolved in solution, in the solid state and adsorbed onto solid particles, like aluminosilicates and magnetic materials, are reviewed and discussed. A short discussion of the thermodynamics and kinetics of the sorption process, with indication of the main mistakes reported in literature, is also given.

## 1. Introduction

Hazardous waste poses an elevated risk to human health and to the environment if it is not managed and disposed of safely. Nowadays, many pollutants, generated from anthropogenic activities, are found in groundwater and surface water, stimulating the search for efficient cleaning procedures. Several techniques have been tested for treating organic and inorganic wastewaters including sorption [1,2,3,4,5,6,7], membrane filtration [8,9], reverse osmosis [10] and, in recent years, advanced oxidation processes, such as photodegradation [11,12,13,14,15,16,17] and electro-oxidation [15,18,19]. Sorption process, a generic term covering the processes of adsorption, absorption and ionic exchange, is one of the most effective methods for wastewater treatment, because of its relatively high efficiency, low-cost, and simplicity [20]. Activated carbons are widely used due to their good sorbing capacity [21]. However, activated carbon is quite expensive and the disposal of exhaust material has a cost for the environment. Recently, many studies have been carried out investigating the sorbent properties of natural and cheap materials [22]. Among them, humic acids (HA), the fraction of humic substances (HS) soluble at neutral and basic pH, appear to be particularly promising. HS are a class of compounds formed during humification, a process of transforming biologically degradable organic matter (mainly plant and microbial constituents) into a stabilized, microbially recalcitrant, sink and source of carbon [23]. HS derive from the association of various components, such as amino acids, lignins, pectins and carbohydrates. Polyphenols coming from lignin probably play a key role in the formation of HS [24]. HS are ubiquitous in water, soil, and sediments, and are of paramount importance in sustaining plant growth and controlling both the fate of environmental pollutants and the biogeochemistry of organic carbon in the global ecosystem [25]. HS are responsible for the structure and physico-chemical properties of soil and are involved in the majority of soil surface phenomena. The current definition of HA is not based on a molecular structure but rather on macroscopic properties, such as the brown colour, the presence of aromatic groups, and an acidic character due to phenolic and carboxylic groups [26]. As the results of stochastic synthesis, HA have a broad molecular weight distribution and high chemical heterogeneity. The main source of HA is leonardite [27], a vitreous mineraloid product formed by natural oxidation of lignite and mined in many countries. Moreover, HS are a large fraction of the organic matter in compost, a soil amendant obtained by the biological aerobic processing of organic wastes [28]. From these materials, HA can be extracted by basic/acid treatment: solubilisation at basic pH and precipitation at acidic pH. HA molecules tend to aggregate in water solution, giving colloids. Self-assembly increases with concentration at a low pH and in the presence of metal ions [29,30]. Because of this, and of the wide mass distribution of HA, an accurate determination of the molar mass of these compounds is a difficult task. Until nearly the end of the last century, it was commonly assumed that HA had a macromolecular structure. Almost every method of molecular mass determination of polymers has been applied to humic acids, including ultra-centrifugation [31], viscosimetry [32], and high performance size-exclusion chromatography [33,34,35]. 

Moreover, some researchers have suggested that HA are not macromolecules, but rather supramolecular assemblies of small units, averaging around 600 g mol^−1^, that are held together by weak intermolecular forces [36,37]. Other researchers have reported the simultaneous presence in the solution of macromolecules and supramolecular assemblies [38]. Recent studies by gel permeation chromatography and long-lasting dialysis experiment using a membrane with selected cut-off have given clear evidence that the HA samples analysed, extracted from leonardite and from compost, have a polymeric structure, and an average molecular weight higher than 8kDa [39].

Studies carried out for several decades have intensively studied the interactions of HA with inorganic and organic pollutants [40,41,42]. Among them, studies regarding the fate of pesticides in soil and sediments are of particular importance for environmental implications [43,44,45,46,47]. It has been widely shown that HA can effectively interact with pesticides, through sorption or covalent bond formation, and thus affect their mobility and transformation in soil and sediments [48,49,50]. A high sorption of pesticides onto soil and sediments is often associated with high HA content [51,52,53]. Pesticides with chemical functionalities similar to HA are those most likely to bind covalently to soil and sediments [44]. HA can reversibly bind cations and non-ionic compounds, including organic pollutants, by means of unspecific interaction processes, such as electrostatic interactions, hydrogen bonds, dipole interactions and hydrophobic effects. More specific interactions between HA functional groups and organic pollutants may also occur. It was shown, for example, that soil and sediment humic acid quinones covalently bind aromatic ammines [54,55] and form stable charge–transfer complexes with electron donor herbicides [56], resulting in the formation of non-extractable residues (NER) of xenobiotics. In addition to covalent bonding, other processes such as a slow sorption and diffusion of the pollutants through the micropores of HA may contribute to NER formation [57]. Given their solubility in water at neutral and alkaline pH, HA are the main carriers affecting the mobility of pollutants in the environment [58,59]. Molecular mechanics and molecular dynamic simulations have shown that HA have a reticulate structure with clear stereo interspaces [60,61]. These interspaces can sorb significant amounts of organic pollutants, which gives HA a good cleaning ability. Molecular modelling showed [62] that in the interaction between polycyclic aromatic hydrocarbons and humic substances, the π–π interactions and H-bonding interactions play an important role. Significant correlations between boiling point and the octanol/water partition coefficient were observed.

In this review, we collect the results of the relevant literature about the sorption of organic pollutants onto humic acids in three different configurations: HA in solution, HA in solid state and HA immobilised onto solid materials as supports.

## 2. Thermodynamic and Kinetic Modelling 

### 2.1. Thermodynamic and Isotherm Models 

At equilibrium, the relationship between the sorbate concentration in adsorbing phase (*q_e_*) and in solution (*C_e_*) is the so-called sorption isotherm, which can be analysed using several models. Some of them start from a physico-chemical analysis of the process. Frequently, because of the heterogeneity of the adsorbent and of the different type of interactions between sorbate and sorbent, these models fail to give a good description of the experimental data and, in that case, it is necessary to use empirical or semiempirical equations for modelling the data [63,64]. The main isotherm models are briefly described below.

The linear model (Henry model): this model requires that there is a constant proportionality between *q_e_* and *C_e_*, condition that can be realized at very low concentrations [65]

(1) $$q_{e}=K_{P}C_{e}$$

where *K_P_* is an equilibrium sorption coefficient.

The Langmuir model: starting from the assumptions of (i) reversible binding between sorbate (*L*) and the sorbing material (*S*) (see Equation (2)), (ii) a limited number of energetically equivalent sorbing sited and (iii) no interactions between sorbed molecules, and considering that at equilibrium the sorbing rate is equal to the desorbing rate,

(2) S +L⇌S·L

the following equation (i.e., the Langmuir isotherm) can be derived [65]

(3) $$q_{e}=\frac{q_{m}K_{L}C_{e}}{1+K_{L}C_{e}}$$

Here, *q_m_* denotes the maximum equilibrium sorption capacity, obtained for *C_e_*→∞ and *K_L_* is the Langmuir sorption equilibrium constant (a thermodynamic equilibrium constant), equal to the ratio between the sorption and the desorption kinetic rate constants. In the plot *q_e_* vs. *C_e_*, the slope at *C_e_* = 0 is *q_m_* × *K_L_*. Although the derivation of this equation is very immediate, it has recently been object of negative criticism [66,67,68].

Protein-ligand like model: this model can describe the binding to dissolved molecules and it supposes that there are *n* equivalent binding sites. Using the mathematical description usually used to describe the binding of ligand to proteins, the following equation can be derived [69]:

(4) $$q_{e}=\frac{n\;C_{e}}{K\;+\;C_{e}}$$

Here *K* is the equilibrium constant of the binding of one ligand molecule on the compound molecule (*CM*) containing already bounded *i* − 1 molecules of ligand

(5) $$CM\cdot L_{i-1}+L⇌CM\cdot L_{i}$$

Temkin isotherm: this isotherm takes into account the effects of indirect adsorbate/adsorbate interactions on the adsorption process; it is also assumed that the heat of adsorption of all molecules in the layer decreases linearly as a result of the increase in surface coverage.

The Temkin equation is an approximation of the Frumkin isotherm for surface coverage values ranging from about 0.2 to 0.8 [70]

(6) $$q_{e}=\frac{RT}{b_{T}}ln\left(A_{T\;}C_{e}\right)$$

where *b_T_* and *A_T_* are the Temkin binding constants. 

Dubinin–Radushkevic isotherm: this isotherm is generally applied to explain the adsorption mechanism with a Gaussian energy distribution onto a heterogeneous surface [71,72]. In the relation

(7) $$q_{e}=q_{m}e^{\left(\beta \;\epsilon^{2}\right)}$$

$q_{m}$ is the maximum equilibrium sorption capacity, *β* is a constant related to the sorption energy and ∈ is the Polanyi adsorption potential $\in \;=RTln\frac{C_{s}}{C}\;$ [73].

The Freundlich sorption equation: this is an empirical model (Equation (8)) particularly suitable for isotherm data which do not reach a sorption-saturated level

(8) $$q_{e}=K_{F}C_{e}^{n_{F}}$$

In this equation, *K_F_* and *n_F_* are the model parameters. This equation has been largely used for describing experimental results, although the constants do not have a physical meaning. The Freundlich model can be mathematically derived for low surface coverage from a Langmuir-like model, which assumes a logarithmic change in the adsorption enthalpy with surface coverage [74].

Regardless of the sorption model used to describe the experimental data, the generic equilibrium constant *K* can be calculated by extrapolating to 0 the plot of *q_e_* vs. *C_e_*

(9) $$K=\underset{C_{e}\to 0}{\lim}\frac{q_{e}}{C_{e}}$$

Some researchers [75] have analyzed the equivalent plot of $ln\frac{q_{e}}{C_{e}}$ against *C_e_*, in this case

(10) $$lnK=\underset{C_{e}\to 0}{\lim}ln\frac{q_{e}}{C_{e}}$$

This method presupposes that at a low solute concentration there is an unlimited availability of empty adsorption sites, and therefore that the adsorption isotherm simulates a partitioning process.

For processes characterized by energetically equivalent sorbing sites and no interactions between sorbed molecules, the *K* value obtained by the extrapolation at *C_e_*→0 is, of course, the same in all the *C_e_* ranges examined. On the contrary, when the sites are not equivalent and/or there is interaction between the sorbate molecules, *K* value is limited only at very low sorbate concentrations [76].

It is well known that useful thermodynamic functions, like the standard enthalpy change (Δ*H*°) and standard entropy change (Δ*S*°), can be obtained by evaluating the temperature dependence of the adsorption equilibrium constant. 

The dependence of the equilibrium constant on temperature (*T*) can be obtained by the van’t Hoff equation [65]

(11) $$\frac{d\;lnK}{dT}=\frac{\Delta H{}^{\circ}}{RT^{2}}$$

where *R* is the universal gas constant.

Integrating this equation under the assumption that Δ*H*° is temperature-independent leads to

(12) $$\ln K=\;-\frac{\Delta H{}^{\circ}}{R}\frac{1}{T}+const$$

where *const* is an integration constant.

This last equation suggests that a plot of *lnK* vs. 1/*T* should produce a straight line, from whose slope Δ*H*° is obtained. 

Moreover, from the relations

(13) ΔG°=−RTlnK

(14) ΔG°=ΔH°−TΔS°

Δ*S*° can be determined.

It is worth noting that the numerical value of *K* (and hence of Δ*G*° and Δ*S*°), depends on the selected standard state [76]. In contrast, Δ*H*° value is not affected by the choice of the standard state because there is a constant ratio between the *K* values at two different standard states.

It is clear from the above considerations that the selected standard states must be always the same in order to correctly compare adsorption thermodynamic parameters from different sources. Moreover, it is necessary to remark that the sign of Δ*G*° gives no information about the spontaneity of the process in non-standard conditions, as is often erroneously reported in literature [77]. The sign of Δ*G*° can depend on the selected standard state [78].

To obtain complementary information to those acquired from studying the equilibrium constant’s dependence on temperature, one can calculate the isosteric enthalpy of sorption. The isosteric enthalpy of sorption (Δ*_ist_H*) represents amount of heat exchanged by the system at constant surface coverage [76]. 

Supposing that Δ*_ist_H* does not change over the temperature and composition range analyzed, the following equation can be used

(15) $$lnC_{e}=\frac{\Delta_{ist}H}{R}\frac{1}{T}+c$$

where *c* is an integration constant. 

From a series of isotherm plots at various temperatures, values of *lnC_e_* for selected surface coverages can be obtained. Afterwards, from plots of *lnC_e_* vs. 1/*T*, values of Δ*_ist_H* for a different surface coverage can be derived, provided that linearity is observed in these plots. A decrease in the absolute value of Δ*_ist_H* with sorption degree may indicate that the sorbent contains sorption sites with a different sorption energy [79,80]. An Δ*_ist_H* which does not vary with the surface coverage suggests that the equilibrium of the adsorption system investigated should be well modelled by a Langmuir isotherm.

### 2.2. Sorption Kinetic Models

Several models can be used to explain the sorption from liquid phase onto a solid phase. Among them, the so-called pseudo-first order, pseudo-second order, intraparticle diffusion (Weber–Morris equation) and Elovich models are the most common.

The rate of adsorption for the pseudo-first order model is

(16) $$\frac{dq}{dt}=k_{1}\left(q_{e}-q\right)$$

Integrating this equation for the boundary conditions *t* = 0 to *t* = *t* and *q* = 0 to *q* = *q* gives

(17) $$ln\frac{q_{e}-q}{q_{e}}=-k_{1}t$$

The pseudo-second-order rate equation is

(18) $$\frac{dq}{dt}=k_{2}{\left(q_{e}-q\right)}^{2}$$

Integrating this equation for the boundary conditions reported above gives

(19) $$\frac{t}{q}=\frac{t}{q_{e}}+\frac{1}{k_{2}q_{e}^{2}}$$

Starting from a reversible binding (Equation (2)), Azizian [81] showed that at very high concentrations of ligand, the experimental data are described by a pseudo-first order rate equation, while when decreasing the ligand concentration the data are fitted by a pseudo-second-order rate equation. The “very high concentration” assumption of Azizian is poorly applicable in adsorption studies because requires that the solute concentration remains virtually constant over time [82]. Moreover, it is worth noting that also nowadays we find papers in highly authoritative journals containing a wrong analysis of sorption kinetic data [83]. A frequent mistake is to evaluate the applicability of the pseudo-second-order model from the linearity of the plot *t*/*q* vs. *t* without excluding adsorption data at (or very close to) equilibrium. The applicability of the pseudo-second order model, or of any other linearizable model, could be evaluated by fitting the data with the nonlinear form of the model and verifying that the residual errors are randomly distributed, as suggested by some authors [84,85].

The Weber–Morris equation [86] is widely used for describing the rate of adsorption governed by intraparticle diffusion of the adsorbate

(20) $$q=k_{D}\sqrt{t}+\kappa$$

Here, *k_D_* is an intraparticle diffusion rate constant and *κ* is related to the thickness of the boundary layer [87].

According to this model, when the plot of *q* vs. $\sqrt{t}$ is linear, the intraparticle diffusion is the rate-limiting step of the adsorption process.

The Elovich model assumes that the rate of adsorption decreases exponentially with time

(21) $$\frac{dq}{dt}=\alpha e^{-\beta t}$$

Here, α and *β* are empirical constants.

An approximated solution of Equation (21) is (supposing that α∙*β*∙*t* >>1) [88]

(22) $$q=\frac{1}{\beta }\ln\left(\alpha \beta \right)+\frac{1}{\beta }ln\left(t\right)$$

The applicability of Equation (22) can be verified by the linearity of the plot of *q* vs. *ln*(*t*) [87].

## 3. Interaction of Organic Pollutants with Dissolved Humic Acids

Rebhun and co-worker [89] reported the removal of hydrophobic contaminants from water using dissolved humic acids as a complexing agent and then adding a flocculant salt (alum or ferric chloride). These salts cause the precipitation and flocculation of HA and of the associated contaminant. The authors studied the removal of polycyclic aromatic hydrocarbons and reported that the proposed process is effective in removing pollutants of medium to high hydrophobicity (log K_ow_ > 4.5).

De Paolis and Kukkonen [90] analysed the binding of benzo(a)pyrene and pentachlorophenol to dissolved humic and fulvic acids extracted from sediments of the Arno river and Tyrrhenian sea. Fulvic acids (FA) are the fraction of humic substances soluble at any pH value. Using relatively very dilute solutions, they showed that HA have a greater affinity for binding hydrophobic compounds than FA. Change in pH only weakly affects the partition coefficients for the neutral chemical benzo(a)pyrene, while the values for the phenol derivative (about 102 times lower) decrease radically with an increasing pH from 5.0 to 8.0. This suggests that only the unionized form of a weak organic acid can interact with the humic material. Moreover, elemental and spectroscopic analysis indicate that a high partition coefficient can be related to a large aromatic content and to a rather low content of functional groups in the humic structures. 

Recently, Leone et al. [91] have analysed the binding of some benzene derivatives (o-xylene, toluene, phenol, and benzyl alcohol) to dissolved humic acids by equilibrium dialyses experiments. The equilibrium dialysis method is widely used for determining the binding characteristic of macromolecules (e.g., HA) versus smaller molecules or ions. A humic acids solution is introduced in a dialyzing tubing with an appropriate cut-off and put in contact with a solution with a composition similar to the inside solution, but without HA and containing the selected sorbate. At the equilibrium, the activity of the diffusible molecules inside and outside the semipermeable membrane is the same, and, assuming an ideal solution, the concentrations are also the same. Material balance analysis gives the partition coefficient. In the study carried out by Leone et al. [91], the HA samples were extracted from compost and from leonardite; for both samples, a macromolecule of HA was observed. The humification index (E4/E6 ratio) and the distribution coefficient between ammonium sulfate/polyethylene glycol solutions showed that the macromolecule of HA from compost has a higher hydrophobicity. Assuming that the binding sites onto HA are energetically equivalent, the binding curves were analyzed, and the amount of ligands bound per unit weight of HA and the association constants were determined according to the Protein–ligand like model. The main results are reported in Table 1.

As can be seen, this study showed that the binding capacity was higher for the HA from compost and for more hydrophobic ligands. Similar results about the role played by the hydrophobic interaction were reported by Mei et al. [92], that investigated the binding of pyrene to HA; they observed that the partitioning coefficients (K_DOC_) decreased by increasing the pH (Table 2).

## 4. Sorption onto Humic Acids in the Solid State

Guo et al. [93] have studied the sorption of tylosin (TYL) and sulfamethazine (SMT), two ionisable antibiotics extensively used in the farming industry as veterinary therapeutic agents and growth promoters, onto solid HA. TYL is a week base, pK = 7.1, then at an acidic pH is a cation and forms an ionic bond with the deprotonated groups of HA; SMT is an amphoteric compound with pK values of 2.28 and 7.42; this implies that the net charge of the compound changes with pH even in acidic medium. The researchers observed that the experimental data, for *C_e_* values lower than 30 mg L^−1^, are well fitted by a linear model and the partition constant decreases with the pH and ionic strength (Table 3 and Table 4).

From these data, the authors deduced that the primary sorption mechanism is the cation exchange between the positively charged sorbate and the functional groups of HA. However, at pH 4 (see Table 4), another mechanism may contribute to the uptake of SMT by HA. It is indeed expected that SMT, when in its neutral form (pH 4), forms covalent adducts with quinones of HA [94].

The sorption of atrazine (pK_b_ = 12.3), a herbicide, onto humic acids was studied by [95]. The adsorption isotherms were well fitted with the Freundlich model and the kinetics data with the Elovich equation. The ionic strength of the atrazine aqueous solutions did affect the amount of the atrazine adsorbed on the substrate, suggesting that, for this compound, electrostatic forces also play a significant role in the adsorption process. At the pH of the sorption experiment (acidic or neutral pH), the sorbate is in cationic form. Some kinetic and thermodynamic relevant data are reported in Table 5 and Table 6.

The values of the parameter n (Table 5, n < 1) and the increase in Δ*_ist_H* with the amount adsorbed (Table 6) clearly show that the sample analysed has a heterogeneity of sorbing sites, to which different energies of interaction are associated.

Seki and Suzuki [96] insolubilised HA by heating at 330 °C for 1 h; analyses of the metal cation complexation showed that two different types of acidic groups were present on HA and the insolubilization process scarcely influenced the metal–complexation constant of acidic groups; however, the number of available metal binding sites on the immobilised HA was decreased to some extent. The solubility reduction was possibly due to a partial loss of –COOH and –OH groups. 

The sorbent properties of insolubilized HA towards some benzene derivatives (toluene, o-xylene, phenol, and benzyl alcohol) were studied by [97]. Samples of calcium salt of humic acids were thermally immobilized following the method proposed by [96]. HA were extracted from green-waste compost (HA_comp_) and from leonardite (HA_leo_), chemically characterized by infrared spectroscopy, carbon nitrogen and hydrogen analysis, ash content, hydrophobicity tests, and molecular weight distribution. The sorbent properties towards the benzene derivatives were studied with the batch equilibrium method. HA_comp_ was found to be less rich in aromatic rings and more hydrophobic than HA_leo_. The maximum amount of sorbate bound at the equilibrium was consistently higher for the immobilized HA from compost than from leonardite and increased with the n-octanol/water partition coefficient (K_OW_) of the adsorbate (Table 7). The data confirmed the prevalent role of the hydrophobic interactions in the humic acids–organic molecules interaction.

Tang et al. [98] analysed the sorption of benzene in function of temperature, pH, and ionic strength. The sorption isotherms showed that sorption of benzene on HA is exothermic and there was no obvious effect of ionic strength on benzene sorption, suggesting that the sorption process is not controlled by ion exchange or electrostatic interactions. The hydrophobic effect and π–π conjugative interaction were the possible sorption mechanisms of benzene on HA. 

The sorption of copper ion (Cu^2+^) and norfloxacin (an antibacterial agent, Nor) onto HA was investigated by [99] under different solution chemistry conditions (pH, ionic strength, and foreign ions). The sorption isotherms were well fitted, with only a few exceptions, by the Langmuir model. The Langmuir parameters recorded in absence of Cu^2+^ in function of pH are reported in Table 8.

This pH dependence is in line with a previous report [100], according to which the primary sorption mechanism of Nor onto HA is the cation exchange.

## 5. Sorption onto Humic Acids–Solid Material Adduct

The observation that HA is an efficient sorbent of hydrophobic pollutants and is also able to interact with aluminosilicates compounds and with some magnetic compounds, has stimulated research for its technical application in the water purification of the immobilization of HA onto solid materials. 

As the size of the final adduct closely depends on the original size of the solid material, by using natural minerals with the appropriate size it is possible to produce HA solid adducts suitable for use in a fixed-bed column, a technology of common application in water purification [101]. Among the most studied aluminosilicates in this field are zeolitic tuffs and mineral clays.

If the adduct is dispersed in wastewater, by using magnetic sorbents there are several advantages, e.g., the magnetic sorbents are easily separable from liquid phase with a magnetic process, a huge advantage in comparison with other materials that require more complex systems for solid-liquid separation. Moreover, the pollutant transfer from water to sorbent is enhanced. 

### 5.1. HA-Zeolitic Tuff Adduct

According to laboratory tests, this adduct is a material particularly efficient for sorption purposes. Many studies have shown that HA can bind to zeolites [42,102,103,104]. It is well known that these minerals are naturally occurring aluminosilicates characterised by high surface areas, high cation exchange capacities and three-dimensional cage-like structures, with channel apertures of a few Angstrom units as order or magnitude. Owing to this structural feature, since their discovery, the zeolites have found wide uses as ion exchangers and molecular sieves [105]. Studies from Capasso et al. [102,103] on the HA sorption onto zeolitic tuffs show that tuff samples enriched by divalent cations took up HA in amounts larger than those observed for the untreated sample, while the opposite was observed for the monovalent cation-enriched samples. It is known that the calcium ion promotes HA coagulation, a rapid binding of Ca^2+^ to HA followed by a slow aggregation [106]. 

A zeolitic tuff-humic acids adduct, useful for environmental applications, was obtained by sorbing humic acids onto zeolitic tuff and then heating the resulting complex at 330 °C for 1.5 h. The size of the zeolite tuff particles used was 0.5–1.0 mm. X-ray powder spectra and IR spectra showed that the crystal structure of the zeolitic tuff and the chemical structure of HA were not altered during their preparation [97]. The thermal treatment induced decarboxylation of HA, thus reducing their water solubility and ensuring their effective immobilization on the tuff. Sorption isotherms were determined at 4, 14, 24, and 34 °C for toluene, cyclohexane, o-xylene, benzyl alcohol, phenol, and cyclohexanol. The data were well described by the Freundlich sorption equation, with the parameter n_F_ of Equation (8) lower than 1. The higher values of the constant K_F_ were obtained for the two aromatic hydrocarbon compounds, toluene (924 mmol^n−1^ L^n^ kg^−1^) and o-xylene (1350 mmol^n−1^ L^n^ kg^−1^). The isosteric sorption enthalpy (Δ*_ist_H*) and the standard enthalpy were negative for all compounds analyzed, and the absolute values of Δ*_ist_H* decreased with increasing sorbate loading, in line with a previous report [79]. The values of the Freundlich parameter n_F_ and the increases in Δ*_ist_H* with sorbate loading indicated the heterogeneity of the binding sites. 

Thermodynamic analysis [107] of the sorption data showed that for the selected hydrocarbon compounds, i.e., toluene, cyclohexane and o-xylene, both the isosteric enthalpy and entropy sorption changes were negative and the process was exothermic. In contrast, for hydroxyl compounds, benzyl alcohol, phenol and cyclohexanol, all the thermodynamic functions were positive; the increase in entropy possibly reflected the release of water molecules during sorption. The opposite signs of the enthalpy change for hydrocarbon and hydroxyl pollutants suggested that a high temperature should favour the sorption of hydroxyl, whereas a low temperature should enhance the sorption of hydrocarbon compounds. By changing the temperature, it is therefore possible to affect a selective sorption/desorption of a class of pollutant. The sorption reversibility of the pollutants studied permits the regeneration and the reuse of the adduct. 

### 5.2. HA-Clay Minerals

It is well known that clay minerals are phyllosilicates and their crystal structure consists of tetrahedral [SiO_4_]^4−^ and octahedral [AlO_3_(OH)_3_]^6−^ sheets in either a 1:1, 2:1 or 2:1:1 proportion. It has been shown that also these natural minerals can bind HA with a good yield [108,109]. Jemeljanova et al. [110] analysed the property of clays-HA adduct to sorb the pharmaceutical chloropromazine hydrochloride. Three types of clay minerals (montmorillonite, kaolinite and bentonite) were coated with three types of humic substances: technical humic acid from lignite, humic substances extracted from raised bog peat and technical K humate from lignite. The results show that clay minerals–humic acids composite materials are good sorbents for the removal of pharmacologically active substances. The sorption capacity of the clay–humic acid composite materials analysed toward chloropromazine is in the range 600 μg mL^−1^ (bentonite–technical K humate adduct) −141 μg mL^−1^ (caolinite–technical K humate adduct). However, it is difficult to compare these results with those obtained with other minerals; the authors did not report data about the size of the minerals used. Sorption onto a mineral-HA adduct strongly depends on the size of the particles.

Jin et al. [111] examined the sorption of Cu(II) and 2,4-dichlorophenol onto bentonite and humic acid adduct. Results indicate that the sorption of either Cu(II) or 2,4-dichlorophenol is little influenced by the presence of the other contaminant. At 30 °C, the amount of sorption was 22.40 mg g^−1^ and 14.23 mg g^−1^ for Cu(II) and 2,4-dichlorophenol, respectively.

### 5.3. HA-Magnetic Materials 

Koesnarpadi et al. [112] reported a study on the sorption of p-chlophenol on humic acids deposited on magnetite. The adduct was obtained by co-precipitation method: a Fe^3+^ + Fe^2+^ aqueous solution was heated at 90 °C, then the pH was brought at 11. Afterwards, HA was added. The average crystallite size was ~10 nm. The sorption of p-chlorophenolon was optimum at pH 3.0 and fitted well to pseudo second-order kinetic models and Langmuir isotherm. For a 20:1 (magnetite:HA) ratio, the q_m_ and K_L_ (Equation (3)) were 0.268 mmol g^−1^ and 1508 L mmol^−1^, respectively.

Xu et al. [113] reported a study on the sorption of pentachlorophenol, an ionisable compound, and phenanthrene, an aromatic, not ionizable, compound, onto nanoparticles of hematite coated with a commercial peat HA and with HA extracted from soil. The sorption isotherms showed that the coating of HA onto the surface of hematite substantially increased the sorption affinity of the magnetic substance for both the analysed sorbents by about 1–2 orders of magnitude, and the increasing degree of sorption was positively correlated to the HA content. The sorption on HA-coated hematite was inhibited with increasing pH values and the pH effect on pentachlorophenol sorption was more significant than that of phenanthrene, due to the deprotonation of functional groups within the adsorbent HA. 

The sorption of some phenols, namely phenol, 4-nitrophenol, pentachlorophenol and nonylphenol, onto Fe_3_O_4_ nanoparticles coated with HA, was investigated by [114]; the main results are reported in Table 9.

The trend of the sorption capacity as a function of the specific HA is the same, and increases with the hydrophobicity of the sorbate. The hydrophobicity parameters for phenol, 4-nitrophenol, pentachlorophenol and nonylphenol are 1.67, 1.61, 4.69 and 5.74, respectively.

### 5.4. Other Materials 

Klavins and Eglite [115] reported the immobilization by the grafting of HA onto different carriers (styrene-divinylbenzene copolymers, cellulose and silica), as well as by crosslinking with formaldehyde. The properties of the obtained immobilised HA were studied, including their potential use as sorbents for several metal ions and organic substances. The sorption isotherms of 4-aminoazobenzene, Crystal Violet, Methylene Green, and flavine mononucleotide onto immobilized HA showed that pH and salt concentration have a significant effect on the sorption process, largely depending on the properties of polymeric matrix [116]. 

## 6. Conclusions

HA are the soluble fraction of humic substances at an alkaline pH and are produced by the biotic and abiotic degradation of dead organic matter. They are a large fraction of the organic matter in compost, a soil amendant obtained by biological aerobic processing of organic wastes. HA show a strong ability to sorb cationic and hydrophobic organic pollutants. Literature highlights that sorbing efficiency is related to the hydrophobicity of the sorbate and that interaction of HA with hydrophobic organics is mainly due to π–π interactions, hydrophobic effect and hydrogen bonding. An overview on organic pollutant removal through HA in three different configurations—HA in solution, HA in solid state and HA immobilised onto solid materials—has been reported. The literature reviewed shows that HA adducts are the first-choice sorbents for organic pollutants’ removal from wastewater. A second outcome of this work is the use of HA bound to natural aluminosilicates or magnetic compounds for effective water depuration from organic pollutants. Moreover, it has been highlighted that several models can be used to explain both thermodynamic and kinetic behaviours of the system, according to the couple sorbate compound and the sorbing material. In any case, it is worth observing that, from organic wastes, it is possible to extract HA that allow the removal of pollutants from water; however, to the best of our knowledge, there are no reports about the application on real systems, a field of research that strongly demands attention in the near future.

## Figures and Tables

**Table 1:** Interaction parameters for the binding of benzene derivatives to dissolved humic acids according to the Protein–ligand like model (adapted from [91]).

Adsorbate	HA from Compost		HA from Leonardite	
	n (mmol kg^−1^)	K (mmol L^−1^)	n (mmol kg^−1^)	K (mmol L^−1^)
Phenol	520	0.0017	680	2.3 × 10^−4^
Benzyl alcohol	940	0.003	1200	0.021
Toluene	7800	0.09	6000	0.09
o-Xylene	18,000	0.11	4900	0.04

**Table 2:** Partitioning coefficients of pyrene binding to humic acids (HA) (adapted from [92]).

pH	K_DOC_ (10^3^ L kg^−1^ C)
4	49
7	30
10	19

**Table 3:** Effect of pH on the partition constant for the sorption of tylosin and sulfamethazine (adapted from [93]).

Tylosin		Sulfamethazine	
pH	K_d_ (L kg^−1^)	pH	K_d_ (L kg^−1^)
3	386	2.5	236
4	352	3.5	216
5	298	5.5	190
7	268	7.5	165

**Table 4:** Effect of ionic strength on the partition constant for the sorption of tylosin and sulfamethazine at pH = 4 (adapted from [93]).

Tylosin		Sulfamethazine	
Ionic Strength	K_d_ (L kg^−1^)	Ionic Strength	K_d_ (L kg^−1^)
0	458	0	258
0.005	424	0.01	216
0.01	386	0.05	177
0.1	375	0.1	154

**Table 5:** Elovich kinetic constants and Freundlich coefficients for the sorption of atrazine onto solid HA (adapted from [95]).

Elovich Parameters		Freundlich Parameters		
α (mg m^−2^ d^−1^)	β (m^2^ mg^−1^)	Ionic Strength (mol L^−1^)	K_F_ (mL^n^ mg^1−n^ g^−1^)	n_F_
700	13.2	0.1	286	0.78
		0.3	299	0.87

**Table 6:** Isosteric heat of adsorption (Δ*_ist_H*) and standard entropy of sorption (Δ*S*°) at different uptakes (Γ), of atrazine onto solid HA at T = 25 °C (adapted from [95]).

Γ (mg m^−2^)	Δ*_ist_H* (kJ mol^−1^)	Δ*S*° (kJ mol^−1^ K^−1^)
0.25	−7.63	59.7
0.75	−1.14	81.5
1.25	1.88	91.6
1.75	3.86	98.3

**Table 7:** Sorption parameters for the selected sorbates onto HA_comp_ and HA_leo_ (adapted from [97]).

Adsorbate	LogK_OW_	HA_comp_		HA_leo_	
		q_max_ (mg g^−1^)	K_L_ (L mg^−1^)	q_max_ (mg g^−1^)	K_L_ (L mg^−1^)
Phenol	1.1	48	2.6	36	4.3
Benzyl alcohol	1.2	74	0.6	47	2.9
Toluene	2.7	110	36.3	82	4.1
o-Xylene	3.1	296	1.0	226	0.8

**Table 8:** pH dependence of the Langmuir parameters for the sorption of norfloxacin onto HA (adapted from [99]).

pH	q_m_ (mg g^−1^)	K_L_ (L mg^−1^)
3.0	171.4	0.134
4.0	526.8	0.017
5.5	513.6	0.017

**Table 9:** Maximum equilibrium sorption capacities for the sorption onto Fe_3_O_4_ nanoparticles coated with HA (average size = 105 nm, specific surface area = 61–77 m^2^ g^−1^) (adapted from [114]).

Fe_3_O_4_ Coated with HA from:	Maximum Equilibrium Sorption Capacity (q_m_, mmol g^−1^)			
	Phenol	4-nitrophenol	Pentachlorophenol	Nonylphenol
Brown coal	0.27	0.67	1.69	1.44
Peat	0.46	0.76	1.81	1.54
Chermozen	0.58	1.40	1.96	1.85
Sapropel	0.61	1.71	2.12	1.96
